# Medication incident reporting in residential aged care facilities: Limitations and risks to residents’ safety

**DOI:** 10.1186/1471-2318-12-67

**Published:** 2012-11-02

**Authors:** Amina Tariq, Andrew Georgiou, Johanna Westbrook

**Affiliations:** 1 Centre for Health Systems and Safety Research, University of New South Wales, Kensington, Sydney, Australia

**Keywords:** Medication management, Incident reporting, Residential aged care facilities, Long term care, Information exchange

## Abstract

**Background:**

Medication incident reporting (MIR) is a key safety critical care process in residential aged care facilities (RACFs). Retrospective studies of medication incident reports in aged care have identified the inability of existing MIR processes to generate information that can be used to enhance residents’ safety. However, there is little existing research that investigates the limitations of the existing information exchange process that underpins MIR, despite the considerable resources that RACFs’ devote to the MIR process. The aim of this study was to undertake an in-depth exploration of the information exchange process involved in MIR and identify factors that inhibit the collection of meaningful information in RACFs.

**Methods:**

The study was undertaken in three RACFs (part of a large non-profit organisation) in NSW, Australia. A total of 23 semi-structured interviews and 62 hours of observation sessions were conducted between May to July 2011. The qualitative data was iteratively analysed using a grounded theory approach.

**Results:**

The findings highlight significant gaps in the design of the MIR artefacts as well as information exchange issues in MIR process execution. Study results emphasized the need to: a) design MIR artefacts that facilitate identification of the root causes of medication incidents, b) integrate the MIR process within existing information systems to overcome key gaps in information exchange execution, and c) support exchange of information that can facilitate a multi-disciplinary approach to medication incident management in RACFs.

**Conclusions:**

This study highlights the advantages of viewing MIR process holistically rather than as segregated tasks, as a means to identify gaps in information exchange that need to be addressed in practice to improve safety critical processes.

## Background

Medications are the most common therapeutic intervention used in residential aged care facilities (RACFs) [1-4]. A medication incident in this context is defined as “any preventable event that may cause or lead to inappropriate medication use or patient harm while the medication is in the control of the health care professional, patient, or consumer. Such events may be related to professional practice, health care products, procedures, and systems, including prescribing; order communication; product labelling, packaging, and nomenclature; compounding; dispensing; distribution; administration; education; monitoring; and use” (p.4) [5]. High medication incident rates in RACFs can be attributed to the high incidence of polypharmacy and changing pharmacodynamics (adjustments in medication selection and dosage in accordance with individual age related and physical changes) of residents [6]. In addition, the presence of cognitive, behavioural, or swallowing problems for some residents may complicate the act of directly administering medications [2] and increase the risk of medication incidents [7,8]. The process of adequate medication management requires appropriate prescription of drugs in accordance with the resident’s condition, communication of instructions regarding dispensing and packaging to the community pharmacy and ensuring administration of the right drugs to the right person, in the right dose, at the right time [9]. In RACFs achieving these objectives requires coordination among all participants in the process. This includes personal carers and enrolled nurses who provide direct care to residents [10], nurse managers who supervise personal carers and resident care plans guided by decisions made by community-based General Practitioners (GPs) who generally are not based at the facility. The medications prescribed by the GPs are supplied by the assigned community pharmacists who are also located offsite [11]. This creates a complex and multi-dimensional environment, which highlights the need for continuity and coordination of care in a way that is different from acute care [12]. Medication incidents encompass any untoward occurrence while the resident is taking or about to take medication [6]. These incidents can occur across any of the above mentioned stages of RACF medication management [13,14].

Medication incident reporting (MIR) offers care providers a means to describe and document incidents that result from system failures [15-17]. It is a core initiative in improving resident safety [18-20]. For RACFs learning from reported medication incidents is a strategy to continuously improve residents’ care and reduce the risk of medication errors [9,21,22]. The aim of MIR is to collect incident information that can allow RACFs to identify and address the safety risks associated with medication incidents by understanding the types of the incidents, their possible or actual effects, and causes [23,24]. Medication incidents in RACFs are monitored by using mandatory or voluntary MIR systems [25]. Most RACFs have paper forms to report medication incidents consisting of mostly narrative free text fields to document the details of the incident [25-27]. This information is then analysed to identify root causes and initiate corrective actions to address incidents [28].

Existing research in aged care has identified poor quality of incident reporting information as a major factor that inhibits continuous improvement in residents’ safety. Gurwitz et al. based on their cohort study of two large long term care facilities (1229 beds) in USA, estimated that 42% of adverse drug events associated with the distribution and use of medicine can be prevented by streamlining existing processes including MIR [29]. Findings from the study indicated that the poor quality of incident reporting in RACFs fails to support sustainable changes and improved residents’ safety [29]. Hansen et al. based on a retrospective analysis of medication incident reports in RACFs identified incomplete reporting, reporting biases and missing data as key limitations of the MIR process, that delay identification of pertinent resident safety issues [16]. Wagner et al. undertook pilot testing of an electronic incident reporting system in US RACFs and identified lack of clarity of reported data and variation in incident documentation procedures as barriers to reliable and efficient incident reporting [16]. Although studies report a high rate of medication incidents in Australian RACFs, there is an absence of studies that offer insight into medication incident reporting practices [30,31]. The limited literature in this domain highlights the poor quality of information presented in MIR reports that impedes long-term improvements in safety.

Despite the growing research attention on patient safety, weaknesses of the information exchange in the MIR process have not been investigated [9,32,33]. It is imperative to analyse the information exchange that underpins the MIR process to identify any gaps in process execution that inhibit collection of quality information [34-37]. The aim of this study therefore was to undertake an in-depth investigation of information exchange in the MIR process in RACFs and identify factors that hinder collection and communication of reliable and timely incident information. The study also offers practical recommendations on redesigning the MIR process to address identified limitations.

### Research setting

Data were collected at three RACFs in metropolitan Sydney, Australia. The selected sites were part of a large non-profit organisation. The community pharmacy, which provides medications for all three sites, also participated in the study. The RACF study sites described their information and communications technology (ICT) arrangements as a mixed (hybrid) system involving paper and ICT. However, all the key medication-related procedures were paper based. The community pharmacy also described their system as mixed; they use ICT for dispensing but still used paper prescriptions and medication charts sent by the RACFs.

**Figure 1:**
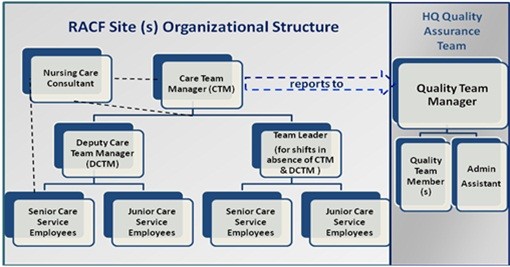
RACF organisational structure.

To investigate the MIR process it was vital to establish an understanding of the organisational structure (Figure 1) of the RACF sites. As the sites are part of one organisation, they had similar operational structures. Each site is led by a care team manager who manages administrative as well as clinical care of residents. The manager is a health professional manager who may also be a registered nurse (RN). When the manager is an RN, he/she can also perform clinical procedures like authorising administration of “when required” medications like pain killers. For the sites where the manager is not an RN, the organisation has an assigned nursing care consultant who is qualified as an RN. The nursing care consultant is only available in-person on the site two or three days a week but available on telephone 24/7 for any urgent circumstances. The manager at each site is supported by a deputy manager, who assists in all the administrative and resident care procedures. The managers are usually present on the RACF site during the morning (8 am - 2 pm) and afternoon shift (2 pm - 6 pm). Team leaders are assigned for the shifts in absence of managers.

Other care staff members in the organisation are known as “care service employees”. RACFs receive pre-packaged medications from their community pharmacy. These are known as drug administration aids (DAAs) [31]. Each DAA indicates the dosage schedule of each resident. Use of DAAs is unsuitable for some medications such as topical ointments, inhalers, and eye drops; therefore these medications are supplied to RACFs as non-packed medications. The care staffs are required to achieve defined competencies in order to be certified to administer medications using the DAAs. At the organisational headquarters the quality management team is responsible for monitoring the quality of the care services provided by the facilities. The quality manager is supported by a qualified team including an RN as well as an administrative assistant to organise and manage information.

## Methods

**Figure 2:**
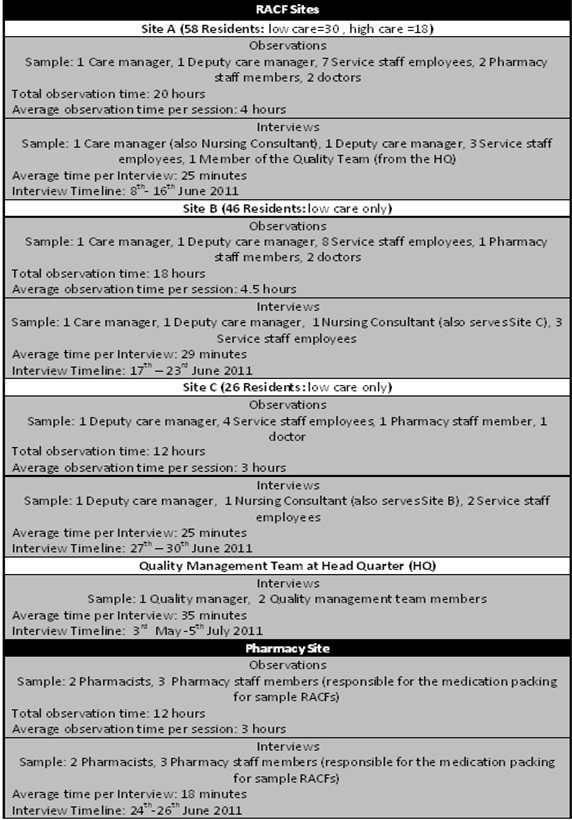
Data collection summary.

The data were primarily collected using non-participant direct observations and semi-structured interviews supplemented with detailed analysis of the artefacts used in the MIR process. One of the authors, experienced in conducting qualitative research in RACFs, [38,39] collected data over three months (May to July 2011) during which 22 days were spent observing work processes. All aspects of the RACF work which related to the MIR process were studied. This included: medication prescribing, ordering, dispensing and delivery by the pharmacy, medication administration and medication monitoring activities. The investigation also focused on the creation and evolution of different artefacts during the execution of MIR activities. Ethics approval for this study was approved by the Human Research Ethics Committee at the University of New South Wales (Reference: UNSW HREC 11115). Written informed consent for participation was obtained from all the participants in the study. Figure 2 presents specific details of the data collection performed across the selected RACFs and pharmacy site.

### Direct observations

Direct observations were conducted of all steps in the MIR process and for the related activities at the community pharmacy. Field notes and photographic images of the artefacts used were the source of data together with some audio recordings of the conversations between RACF and community pharmacy staff. The staff observed at the RACF included care managers, nursing consultants and care service employees who are actively involved in the medication administration process. Observations were conducted during day shifts (7 a.m. to 3 p.m.), as the medication related activities are more intense during this time, and included at least two rounds of medication administration (breakfast and lunch). During observations specific questions were asked to clarify information about the MIR process and the related use of artefacts. This served as a valuable data source enhancing our understanding of domain complexity, and revealing issues not identified by observations. The observations also guided the preparation for follow up interviews. Data collection therefore was mainly driven by first observing the events, the subsequent interviews with the participants to gain insight into the context of activities and reasons for the associated information exchange. Observations were also supplemented with artefact analysis. Artefacts in this study encompass “any artificial device designed to maintain, display, or operate upon information in order to serve a representational function” (p.17) [40]. All the key artefacts used in the MIR process, namely incident report forms, site specific monthly summary charts, key quality indicator (KQI) forms and quarterly organisational incident reports were analysed to determine their information content, physical form, representations used (e.g., symbols, abbreviations and titles for the artefacts) and inter-dependencies.

### Semi-structured interviews

The selection of interviewees was driven by purposive sampling methods - based on their involvement in the medication process in the RACFs. All interviews were conducted in the workplace locations including RACF staff rooms, care managers’ offices and the medication packing room at the pharmacy. This allowed participants to demonstrate how they carry out tasks related to their job. The RACF staff members including the managers and the care staff were asked detailed questions on a range of issues about information exchange relating to MIR, the different activities and artefacts (the context of use, structure and content) communication and coordination with other professionals involved in the process, as well as any information-related issues they experienced in the MIR process [See Interview Protocol in Additional file 1]. Selected members of the quality management team were interviewed to gain insight into how MIR information is used at the organisational level. All interviews were audio-taped, professionally transcribed and verified by one member of the research team checking the accuracy of the transcript with the audio.

### Data analysis

We used thematic analysis based on a grounded theory approach to derive themes relating to information exchange in the medication incident reporting process [40]. The rationale for adopting grounded theory was its ability to guide the exploratory nature of the study and allow emergence of theory from the data [41]. The analysis was carried out with the help of qualitative analysis software NVivo [42]. The data from interview transcripts, observation data and notes from the artefact analysis were triangulated and iteratively analysed over a multi-phase process [43]. Triangulation of data suited the study design, as the combination of interview and observational data allowed in-depth understanding of the key processes involved [44,45]. Analysis phases included a) open coding, in which text passages in the interview transcripts and field notes were examined for recurring themes and ideas; and b) axial coding, in which themes were organised into meaningful relationships. One of the authors performed the initial open coding of the data for content pertaining to the description of the different stages of the medication incident process. This included an analysis of how information about the incident is communicated internally across the facility and externally to participants like GPs and community pharmacists. The analysis also investigated how the information is collated and presented to the quality management team at the headquarters. The initial coding was then shared with the rest of the authors and was reviewed to identify the need for any restructuring of the coding scheme and as a basis for planning subsequent interviews and observation sessions [46]. The data were iteratively analysed in regular research team meetings in order to identify emergent themes, to clarify inconsistencies or unusual findings. For validation purposes the analysis was also presented to selected study participants who confirmed the findings.

## Results

This section begins with an in-depth description of the dynamics of information exchange in the MIR process, followed by a discussion of existing gaps in the information exchange that underpins the MIR process based on the triangulated analysis of the qualitative data (Figure 2).

### Key artefact: Medication incident report (MIR) form

**Figure 3:**
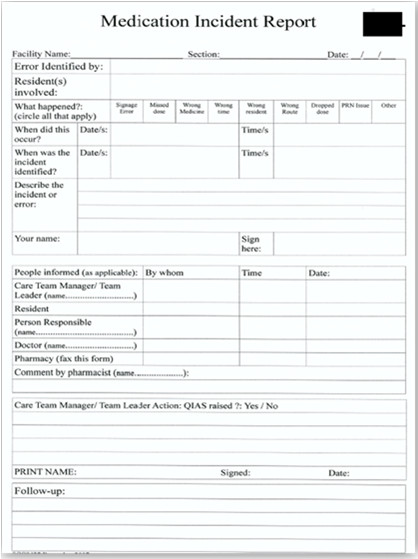
Medication Incident Report Form.

**Figure 4:**
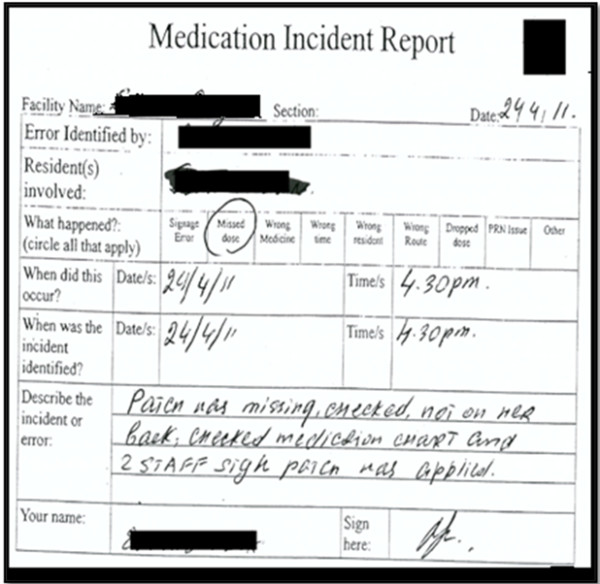
Section of MIR form filled after incident identification.

Medication incidents are reported at sites using the organisation’s standard MIR form. The MIR form is based on the guidelines provided by Australian Pharmaceutical Advisory Council (2002) for RACFs. However as the RACFs can customise the form to their requirements, there might be slight variations regarding medication incident documentation between different organisations [19]. Artefact analysis revealed that the MIR form in the study organisation is a combination of structured checkbox-type fields, as well as some free text fields used to describe the incident (Figure 3). The key data elements captured in the incident report form can be categorised into four groups. The first includes fields which capture the date of incident identification, details of the staff that identified the incident and the name of the resident involved. The second set of fields focus on the incident including type and time of occurrence followed by a free-text description of the incident (Figure 4).

The third set of fields collect information related to the immediate response to the incident. The name of the doctor attending the resident is recorded along with the details of who contacted the doctor (if he/she was informed). Based on the type of incident, the report may also require information about whether the community pharmacist was informed. If the report was faxed to the community pharmacist, his/her comments on the incident need to be recorded on the same form. The final group of fields on the MIR form are filled in by the manager and relate to the quality assurance actions taken in response to the incident and how the manager followed up those actions.

### Medication incident reporting process

**Figure 5:**
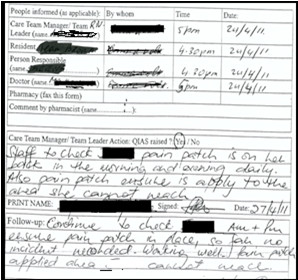
Section of MIR form filled follow-up actions.

This section describes the flow of the MIR process across the study sites. Triangulated qualitative data facilitated the identification of the flow of activities during the process of medication incident reporting at the sites and headquarters. The MIR process in the study sites relies on staff or care managers to identify incidents and submit a MIR form. This can be done during regular shifts. If a staff member identifies a medication incident, he/she has to inform the manager as explained by deputy manager site A “If there’s an issue that was identified staff have to fill in the form, but before that they will have to let manager know, every incident whether it’s a medication or regarding medications everything is informed to [the] manager”. Depending on the seriousness of the incident, the manager may decide to inform the doctor or nurse consultant. For the evening and night shifts, the team leader contacts the manager by telephone to seek guidance on the actions to be taken. Managers who may not be on the site sometimes contact doctors directly to report the incident. Staff members or manager(s) complete initial sections of the MIR form after taking actions to deal with the incident (Figure 4). As explained by the care staff at site C “We have to write down the dates, the time, what happened, how it happened”. The partially filled report is then forwarded to the manager or team leader. They then document details of how the incident was managed and which stakeholders were consulted in response to the incident (Figure 5). As explained by care staff member at site A “The manager will document the follow up, like what has been done for this mistake. What action we have been taken, like [for example] staff education”

Communication with the pharmacy is done only when incident is related to them (such as a drug packing error). As informed by deputy manager site B “We do not need to communicate with pharmacy for incidents all the time, only if there is a need, if there is a query about a medication, or there is a medication missing”. The manager or team leader needs to fax the partially filled MIR form to the pharmacy to get their comments on the incident. The manager or staff member also needs to update the resident’s electronic progress notes to ensure that incoming staff are aware of the incident. The organisation has a pre-established set of rules which help the manager identify when a medication incident involves a quality assurance issue. The last part of the report focuses on documenting information when the incident led to a quality assurance issue. This requires free text description of what actions were taken to manage the issue and how the actions were followed up (Figure 5). The MIR report is completed only after the quality assurance actions have been executed.

**Figure 6:**
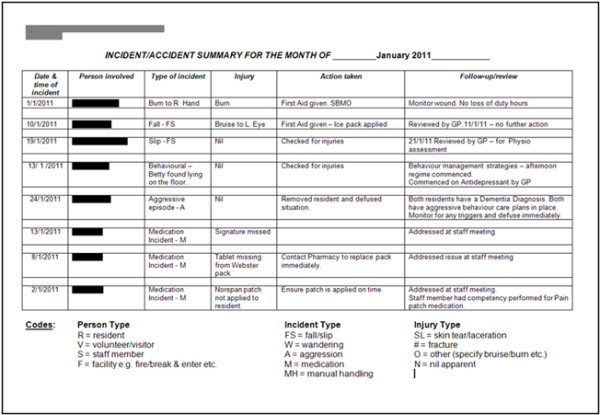
Site specific monthly incident report.

**Figure 7:**
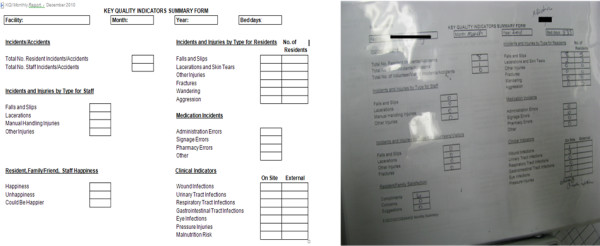
KQI Form (including number of medication incidents.

The completed MIR forms are held in temporary storage files by the manager who then prepares monthly summary reports (Figure 6) which document the type of incidents which have occurred. The managers are also required to complete a monthly key quality indicator (KQI) form (Figure 7), which includes the number of medication incidents of different types. The monthly summary reports and KQI forms are either faxed or emailed to headquarters. At headquarters the quality management team utilises the monthly summary incident reports from sites and the KQI forms to generate organisational level incident summary reports. The files received are managed by the administrator who also prepares an Excel file which is analysed with support of Excel experts (part-time). The quality team manager then writes a narrative quarterly report to explain the trends in the collated data. The report prepared by headquarters is shared with the organisational board’s quality committee and the individual sites.

### Latent gaps in medication incident reporting process

**Figure 8:**
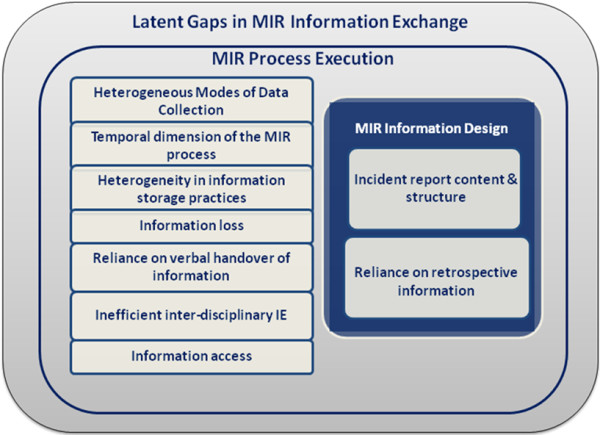
Latent gaps in MIR information exchange.

Themes identifying latent gaps in information exchange that impact on the quality and reliability of the collected information in the MIR process were identified. These themes were grouped into two broad categories based on their relevance to different dimensions of information exchange identified in the analysis (Figure 8). The first category of themes relates to the gaps in the information design i.e. the content and structure of the key artefacts intended to collect incident information. The second category focuses on information exchange during execution of the MIR process. These thematic categories are intertwined and overlapping, as the existence of one gap may lead to another gap.

#### MIR information design

There are no specific national guidelines for RACFs directing when a medication incident report should be filed or what data elements should be included in the incident forms. The organisation in our study had customised a report form based on their organisational policy, accreditation requirements and national pharmaceutical guidelines [47]. An analysis of the artefact templates and their process of creation during the MIR process revealed gaps in the content and structure of the information collated in the process which include:

##### Incident report content & structure

**Figure 9:**
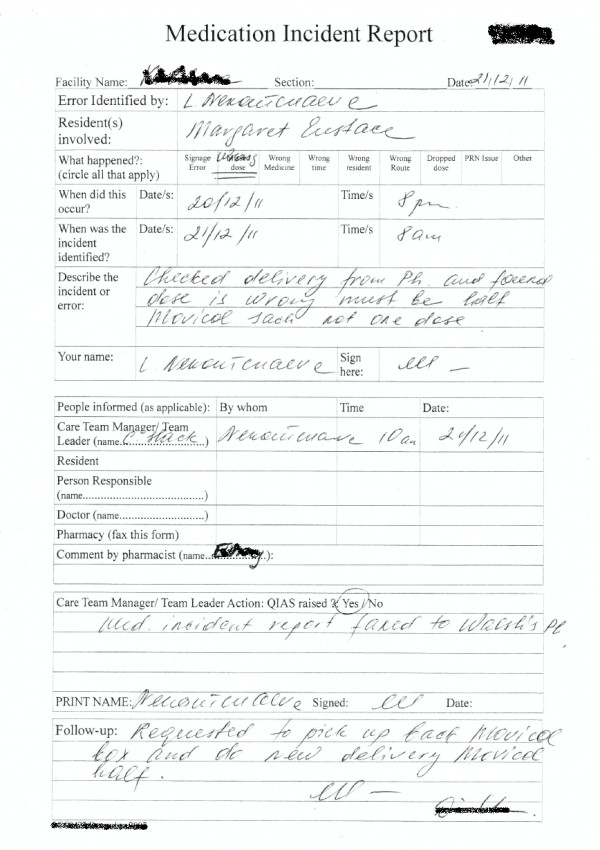
Sample MIR report (with free text and annotations.

The MIR data collection process focuses predominantly on gathering a description of the incident as it unfolded. It contains limited information about any causal or contributory factors that led to the incident and whether there were any features in the system that are available or that are needed to prevent future incidents. For instance was it a charting error that led to the missed dose incident, a communication problem that resulted in a packing error or a labelling error that lead to wrong medicine administration? Also the occurrence of one incident may lead to simultaneous occurrence of another. For instance a missed signature can also result in a missed dose error; but if the dose has been administered then it will only be a missed signature. As explained by a care staff member at Site A: “Most common are just the committing [of]… signage errors. Mostly staff may have kept [the medication] trolley at one place [the RACF corridor or medication room] and they go to the [resident’s] room to give the medicine and they may tend to forget to sign [when they come back from the room]. Similarly a missed dose might be identified despite the fact that its administration has been signed by the staff as informed by care staff at site C “If the medications haven’t been popped out from the pack, I can find out that the medication hasn’t been given to the resident. So I can find out the staff … who have been rostered on that particular time and signed the sheet. For non-packed medication it’s difficult to identify missed doses”. This connection between error types is not clearly captured by the present MIR form which discretely categorises errors. At present there is no record of the impact of the incident on the resident such as whether it was minimal, severe or required a hospital admission. Besides the absence of important information, MIR forms mainly consist of handwritten, free-text information which is difficult to read and interpret as shown by the MIR report in Figure 9. This makes it harder for the manager to decide how much and what information needs to be transcribed into the monthly summary report forms. Managers have to spend a considerable amount of time tabulating monthly incident summaries as it requires them to summarise the narrative details presented in the MIR forms.

##### Reliance on retrospective information

The integrity of the reported information is a major concern in the MIR process because of some key limitations related to retrospective data capture. For instance, the information in the medication signing sheet (used for recording administration) is not locked after an administration session is completed. This allows staff to alter or sign the sheets if they forgot to do earlier. As observed at Site C, a staff member did both breakfast and lunch time medication administration. During lunch time administration she noticed a missing signature on the signing sheet for the breakfast time for one of the residents. She recalled that she had administered the medication and signed for the breakfast time at lunch. The deputy care manager (Site A) also reported that the most common incidents were related to staff administering medications and then realising that they have not signed the administration sheet but do so afterwards. This inaccuracy of information was recognised by the quality team manager who stated that “We do generate the signage errors but those figures aren’t as accurate as we would like them”. The impact of inaccurate retrospective data may result in limited improvements in residents’ safety both at the site and organisational level. This process also contains no information on how long it took the site to manage the incident and complete the MIR report as indicated by the sample of the MIR artefact in Figure 9.

#### MIR process execution

Besides problems in the content and structure of the artefacts, our analysis also highlighted gaps in the execution of the MIR process that limit the reliability of the collected information and obstruct its seamless execution.

##### Double handling of information

**Figure 10:**
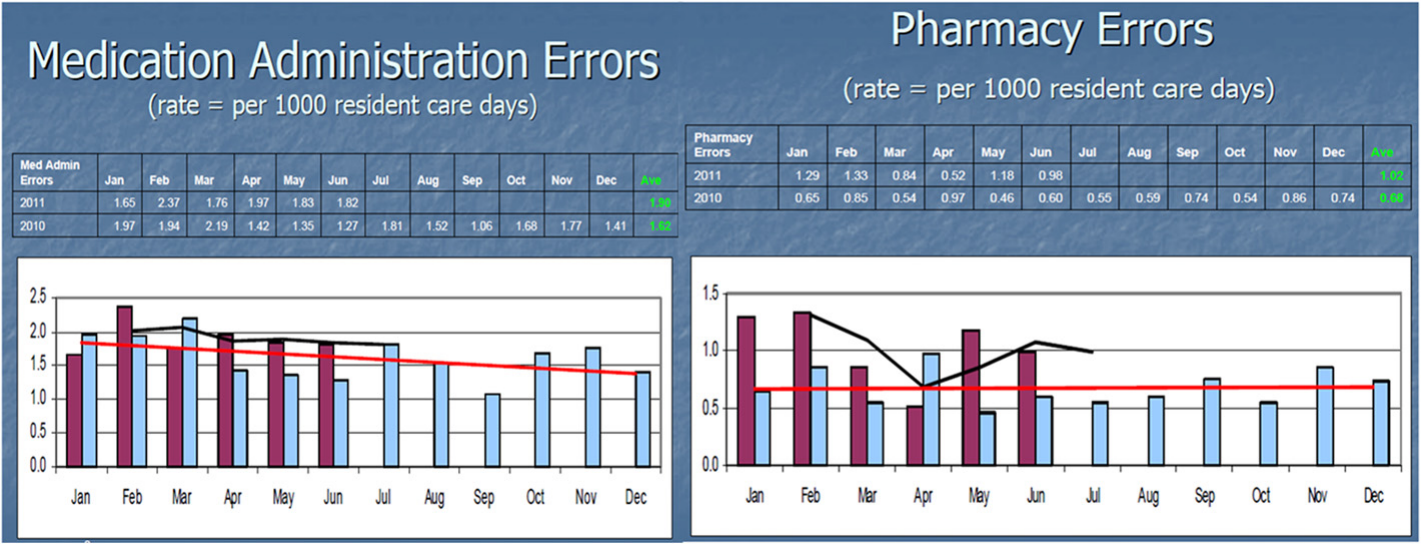
Analysis reports prepared at the headquarters.

Double handling of information occurs at various stages in the process. This occurs when information collected on the MIR form is required to be updated in the electronic progress notes. As informed by one of the care managers (site C) “I have to fill in this incident report, I have to write in progress notes as well, as everybody can go to progress notes and read progress notes to know about the incident”. As observed when preparing monthly incident summary reports double information entry occurs when information first collected on MIR forms is transferred to the site specific summary forms and also used to fill in the monthly KQI form. A similar description of information double handling was given by the deputy care manager (site B) who said that: “Every month the manager does the summary incident report for incidents and writes it all up and faxes it to headquarters. She counts and manually enters in the KQI form using information from the MIR forms”. The sites can send the monthly incident summary and KQI forms in paper only or electronic only or hybrid (one electronic and the other paper) form. Artefact analysis and observations revealed that reliance on these heterogeneous modes of information exchange require headquarters to undertake extra steps to collate information and to generate the graphical views of the incident data (Figure 10). As explained by the quality team manager “My admin support person prepares that data for me and I just monitor. Data entry [is] done by the admin person into Microsoft Excel based on information obtained from sites”. The manual transfer of information from paper to electronic, or from paper to paper at all these stages increases the risk of transcribing errors.

##### Temporal constraints in MIR completion

Time constraints represented an important barrier to MIR in the RACFs. The time from identification of an incident to reporting may be delayed because staff are busy. As one care manager said “My staff needs to be with the residents all the time when on duty. They cannot do all the documentation and sit on the computer for long hours. Therefore I sometimes give them some time to complete the incident reports”. It was observed that the time taken to complete the report also depended upon how rapidly contact is established with the required stakeholders like community pharmacists or GPs.

##### Heterogeneity in information storage practices

Sites have different ways of storing MIR forms. It was observed that at one of the sites the manager does not put a copy of the incident form in the residents’ file despite the designated place in the residents’ paper based files for incident reports. It is vital to place MIR forms in the residents’ files as they are used during care plan reviews and may prompt the nursing care consultant or the GP to revise the medication regime of the residents. However as informed by the deputy manager site (A) “We keep incident forms in a different folder and do not put them in resident files as it makes it easy to have them in one place to prepare the monthly summary reports”. In contrast at site C forms are placed in the residents’ files as informed by the manager “We put the [completed] incident forms… in the resident’s file”. The absence of incident forms in the residents’ folder can cause delays in necessary care plan revisions which can risk residents’ safety. The partially completed incident reports are usually required to be given to the manager. However if the manager is unavailable, the temporary storage of these forms is disorganised as explained by a deputy manager (site B) “We found the safest way for handing in the incident reports when managers are not there is if there’s a incident at night when we’re not here, is to slip it under [the] manager’s door otherwise it could get misplaced if they leave it around reception. It’s the safest way unfortunately, plus it’s for privacy too if visitors come in, or family, they may see it just lying around out there and read it and they shouldn’t be able to read anything like that”. The quality team member also explained the persisting heterogeneity of storage practices in individual sites “There should be a copy in their resident’s file. Its site specific… each team might store theirs differently. Some might have all their medications incidences in a file; it’s usually the site manager who decides how to store the copies of the forms”. The heterogeneity of storage practices can lead to missing MIR reports at the time of monthly report preparation which can result in missing important information in the monthly summary reports and KQI forms.

##### Information loss

The preparation of the monthly incident summary form requires managers to select information from the MIR forms. This may result in instances of information loss as it is up to the manager to decide what to place in the summary report. Headquarters has no direct access to the individual MIR forms and any instances of missing important information at this stage cannot be identified by them. Any incidents such as the manager forgetting to enter information of a particular incident cannot be tracked in a timely way. At the organisational level the availability of increasing volumes of data necessitates headquarters to be selective in their analysis of site level information. As informed by the quality team manager “We don’t graph all of those errors. We don’t standardise all of that stuff. We could do that but you could end up spending all the time just doing a whole lot of information so we just, as an organisation, we decide on which are the – which of the KQIs we want to do and so it’s been administration and pharmacy errors.”

##### Reliance on verbal handover of information

To convey the information regarding the incident, the sites mainly rely on verbal handover by the manager to the incoming staff. For example a care staff member (Site A) said “Usually the manager or deputy manager will let us know what happened [and] follow up. They’ll talk us through what happened … but that could be the next shift or whatever”. The sites in this study do not necessarily have formal hand over meetings which increase the responsibility of the manager to ensure that MIR information is appropriately conveyed to all incoming staff.

##### Inefficient multidisciplinary information exchange

In the existing MIR process it’s at the discretion of the managers or team leaders about whether or not to inform doctors or the community pharmacy. The clinical name of the medication or specific clinical perspective of the incident is not recorded in the MIR form as RACF staff are not qualified to report such information. It is therefore the decision of the manager to exchange information with the clinical experts like the community pharmacist or GP to identify any clinical patterns/reasons leading to the occurrence of the medication incidents. During the study the instances of immediate contact to doctors in case of medication incidents were very few and most of the time it was the manager or the nursing consultant who decided on the course of action in response to the incident. In the case of a packing error from the pharmacy which requires medication repacking as well as comments from the pharmacy on the MIR form, information exchange mainly relies on fax and telephone. As explained by a deputy care manager (Site C):“I do an incident report for that and ask pharmacy staff responsible to sign it by faxing the form filled to them for signature and also ask them maybe by just calling to give their comments on incident if they want to”.

##### Information access

Timely access to information is an issue when the manager or nursing consultant is not available on site at the time of incident occurrence. During these shifts the team leader takes the responsibility of deciding upon the required response to incidents. In scenarios like a missed dose incident the manager or nursing care consultant needs to be informed about the incident; however they do not have access to resident medication records. As the nursing care consultant (Site B & C) said “I have access to IT system 24/7; if all the information is put in to the system then I can just bring that up and look at [the] diagnosis, look at the recent notes. I don’t have access to the medications. I have instructed staff that if they need to call me for medication reasons, they need to have … the medication chart with them so they’re not running to and fro and making excessive phone calls”. Remotely dealing with incidents may lead to an inappropriate decision or delay the decision until the manager arrives on site. This limited access to information therefore obstructs the timely execution of the MIR process.

## Discussion

This study demonstrates how in-depth analysis of the MIR process based on evidence from multiple data sources can provide critical insights into the design of information exchange and its execution in RACFs [13,48]. The findings highlight three information exchange dimensions that need to be addressed to improve MIR process. The first relates to the need to design artefacts that enable the identification of the root causes of the incidents; the second to the need to improve the execution of the MIR process by facilitating seamless flow of accurate information within RACFs; and the third to the need for proactive exchange of information among key stakeholders to encourage a multi-disciplinary approach to medication incident management in RACFs.

The RACFs in this study used custom designed artefacts to meet their organisational requirements. The key limitations in the design of the incident report artefact were an absence of information on the cause and implications of the incident, a reliance on free-text information and the use of retrospective data. There is agreement in the literature about the need to report incident data in a manner that can lead to identification of root causes without increasing the time to report [1,24,49-51]. This requires careful redesigning of MIR artefacts to eliminate unnecessary narrative information which can impose a burden on the time taken to report and may also result in reporting bias [18,52]. To improve the integrity of the data and reduce subjectivity bias in the retrospective data collection it is recommended to design artefacts with standard checklists [53]. Studies in other settings like hospitals have designed MIR forms with checklists of the common breakdown points in the process that lead to the occurrence of the medication incident [27]. Peker et al. identified a similar approach to reporting as effective in documenting resident outcomes [54]. The collection of such information can allow the organisation to analyse the interrelationship between different factors that lead to medication incidents [53]. For example, the cause of an incident during medication administration might be directly related to the other stages of medication management [55]. There might be a labelling issue during its packing or an information handover issue during shift change that leads to an incident during medication administration [11,22,56]. The MIR report therefore needs to capture this path of incident occurrence to provide an illustration of the interdependencies between different factors that lead to incidence occurrence [52,53]. This may improve the effectiveness of reporting processes in RACFs by facilitating actions that progress residents’ safety [18,22,50].

Besides improving the design of the artefacts, integrating the MIR process with the organisational information and communication technology (ICT) system can address several information exchange issues in MIR execution [25,51,57]. As indicated by recent studies, a well-designed, integrated and carefully implemented electronic reporting system can have several advantages including accessibility, increased accuracy, elimination of illegible forms, ease of use and automated reporting [15,18,28,58]. Use of electronic artefacts based on standard computerised spreadsheet formats, supported by well structured dropdown selection functions and self populating fields may reduce the report creation time at the sites, mitigate information storage issues and facilitate timely collection of data by the headquarters [26,59,60]. At site level this possible automation of the MIR documentation process can free up RACF staffs’ time for more direct care [61]. At headquarters, the creation of monthly and quarterly reports can be improved by an ICT system with pre-defined report templates, built-in queries which can be exported to different reporting tools to generate the graphical views [18,48,62]. This would alleviate the need of external help for generating graphical views and also avoid possible transcription errors due to double handling. In an electronic MIR system computerised incident alerts can be used for timely handover of information to incoming staff across shifts [63,64]. Automated recording of process efficiency measures such as total response time to the incident and the tracking of changes to the report can help the organisation-wide evaluation of the rigour in the MIR process. With recent advancements in the use of handheld devices for point of care reporting, the organisation could consider providing staff with handheld devices to further reduce any delays in incident reporting as part of their continuous improvement initiatives [51,65,66].

The third key aspect that the study findings draw attention to is the limited information exchange between RACFs, community pharmacists and GPs [19,49,67]. The multi-dimensional nature of medication management in RACFs demands a holistic approach to incident management by exchanging required information with all the stakeholders [27,32,68]. Proactive sharing of incident information with the community pharmacists and GPs can be achieved by the inter-organisational integration of ICT systems [13,18]. In the absence of electronic systems in the external organisations (GP clinic or community pharmacy) or because of interoperability issues, automated alerts can be sent on the pagers or mobile devices of the concerned stakeholder [55]. This electronic exchange of information at the inter-organisational level can minimise double handling, reduce delays associated with paper based fax procedures and encourage a multi-disciplinary approach to incident management [25]. The data reported in MIR can lead to improved in-depth analysis to detect and mitigate the inevitable medication incidents due to breakdowns in the collaborative nature of medication management [69].

Residents in aged care facilities have extended stays which may span many years and multiple medications (on average six to seven a day) [23]. This situation mandates a longitudinal approach towards resident care together with well-defined procedures that can allow organisations to learn from any incidents or adverse events [9,48,66]. The complexity of medication delivery systems in the RACFs combined with human factors suggests that an error-free medication management environment is probably unlikely [70,71]. Nevertheless, continuous learning from system failures can minimise the factors that lead to the occurrence of medication incidents. Streamlining the incident reporting process therefore can reduce safety risks and allow the active engagement of the quality management team with pharmacists and GPs to establish multidisciplinary approach to incident management.

### Limitations

This study was limited to one organisational setting with distinctive characteristics including the use of organisational specific artefacts. The in-depth analysis of the MIR process was presented to the domain experts and analysed iteratively as a key validation procedure to enhance the applicability of the findings across different RACF settings. This study did not measure the actual time taken to execute different stages of the MIR process and variation of practice in reporting different categories of incidents. Future studies focusing on the use of ICT as an intervention and comparing the results with the traditional paper based procedures based on performance measures such as time spent on key activities can provide evidence of how and where ICT may improve MIR process in RACFs.

## Conclusions

To our knowledge this study is one of the very few that have explored the complexity of information exchange in RACFs’ MIR process. The in-depth investigation, using data collected from multiple data sources enabled identification of latent gaps in the MIR process. This study identified pertinent limitations in the design of artefacts and MIR process execution which need to be addressed to improve the reliability and effectiveness of the MIR process. Redesigning artefacts to capture the cause of incidents and well planned integration of the MIR process with the existing ICT system, can address these gaps in information exchange. The findings from the study emphasise the need for an effective MIR process that supports incident reporting but also facilitates learning from those incidents to improve residents’ safety.

## Competing interests

The author(s) declare that they have no competing interests.

## Authors’ contributions

All authors were involved in the analysis and interpretation of the data. AT/AG/JW were involved in the conception design and data collection of the data. Both AT and AG drafted the paper. All authors were involved in the interpretation of the results and the critical revision of the paper. All authors were involved in the paper’s final approval before submission.

## Pre-publication history

The pre-publication history for this paper can be accessed here:

http://www.biomedcentral.com/1471-2318/12/67/prepub

## Supplementary Material

Additional file 1 Appendix A-Leading Question. Click here for file
