# Supplementary material for: Medication incident reporting in residential aged care facilities: Limitations and risks to residents’ safety
Source: BMC Geriatr. 2012 Nov 2;12:67. doi: 10.1186/1471-2318-12-67 (PMC3547703; doi:10.1186/1471-2318-12-67)
Supplement: Additional file 1 — Appendix A-Leading Question. [file 1471-2318-12-67-S1.pdf]

Appendix A- Leading Questions- Semi-Structured Interviews

For RACF staff and managers

1. Please introduce yourself and your role as a care provider in the facility.
2. Describe the areas of your work/activities you need to perform with relevance to the medication management of the residents in the facility?
3. Can you briefly describe how medication incidents are reported at your facility?
4. Can you explain which information is recorded on the medication incident forms?
5. Can you provide examples of how you go about storing the medication incident forms?
6. Can you provide examples of how you go about reporting for different type of medication incidents?
7. How do you communicate regarding medication incidents with the doctors?
8. How do you communicate regarding medication incidents with the pharmacy?
9. For Care Managers:
    a. How do you follow up on the incidents with the staff?
    b. How are the incidents reported to the HQ?
    c. How do you get feedback from the HQ?
    d. How is the feedback (if any) from the HQ implemented?

For quality management team at Headquarters

1. Please introduce yourself and your role as a member of the quality management team in the organisation.
2. Can you briefly describe how medication incidents are reported in your organisation?
3. Can you describe the policies/ organisational protocols in relevance to medication incident reporting?
4. Can you describe how medication incidents are recorded in your facilities?
5. How do the sites communicate information related to medication incidents to the HQ?
6. How do you communicate with pharmacies and doctors regarding medication incidents?
7. Describe the activities performed at the HQ to analyse the incident data received from the sites?
8. What are the key contents of the incident report prepared at the HQ?
9. How the team at HQ does provide feedback on medication incidents to sites?
